# WORRIES OF OLD-AGE SUPPORT FOR PARENTS AND THEMSELVES: VIEWS FROM OLDER BACHELORS IN RURAL CHINA

**DOI:** 10.1093/geroni/igz038.1710

**Published:** 2019-11-08

**Authors:** Ying Wang, Huijun Liu, Bei Wu, Yaolin Pei

**Affiliations:** 1 Xi’an Jiaotong University, Xi’an Shaanxi, China; 2 New York University, New York, New York, United States

## Abstract

Due to China’s gender imbalance, it is estimated that more than 30 million adult males were unable to get married. The old-age support for older unmarried sons (so-called forced bachelors) and their parents faces a significant challenge. Using data from a survey in Central and Western rural China, the present study examined the impact of family structure and health status on the worries about old-age support for themselves and their parents from the perspective of older unmarried sons. The sample included 359 older unmarried sons with rural Hukou (housing registration) status. The age of he sample ranges from 28 to 51. The results showed that 52.64% and 54.8% of respondents were worried about their own and their parents’ old-age support, respectively. Ordered logistic regression showed that having a sister(s) was negatively related to worries about their own and parents’ old-age support. Those with living mothers had less worries than their counterparts, and those who had a brother(s) had less worries about their parents’ old age support. Moreover, having any brothers who were also older unmarried sons was positively related to worries about their own and parents’ old-age support. Older unmarried sons who had two frail old parents had more worries for their parents’ old-age support than those whose parents were physically independent. The study highlights the importance of family structure and parental health status as important factors in worries over old-age support in China.

